# Sleep quality and nocturnal pain in patients with femoroacetabular impingement and acetabular dysplasia

**DOI:** 10.1186/s12891-020-3151-6

**Published:** 2020-02-28

**Authors:** Nisha Reddy, J. Riley Martinez, Edward Mulligan, Paul Nakonezny, Joel Wells

**Affiliations:** 1 Department of Orthopaedic Surgery, University of Texas Southwestern Medical Center, 1801 Inwood Rd 1st floor, Dallas, TX 75390 USA; 2 Department of Population and Data Sciences (Division of Biostatistics), University of Texas Southwestern Medical Center, Dallas, Texas 75390 USA

**Keywords:** Pittsburgh sleep quality index, Sleep quality, Hip dysplasia, Femoroacetabular impingement syndrome

## Abstract

**Background:**

Femoroacetabular impingement (FAI) syndrome and acetabular dysplasia (AD) are common pathologies that lead to pain in the young adult hip. Nocturnal pain in these patients is often reported, yet little is known regarding the effect of these hip pathologies on overall sleep quality. The purpose of this study was to evaluate sleep quality in patients with AD and FAI syndrome.

**Methods:**

This cross-sectional study consisted of 115 patients who complained of hip pain secondary to either FAI syndrome or AD. One hundred fifteen patients with hip pain secondary to FAI syndrome and AD were assessed using the Hip Outcome Score (HOS), Modified Harris Hip Score (mHHS), and then Hip disability and Osteoarthritis Outcome Score (HOOS). Sleep quality was assessed using the Pittsburgh Sleep Quality Index (PSQI). Multiple linear regression, with adaptive LASSO variable selection, was used to assess factors associated with sleep quality.

**Results:**

Of the 115 patients, 62 had a diagnosis of FAI syndrome and 53 with AD. The mean age was 34.55 ± 11.66 (age range: 14 to 58 years), 76.52% had an ASA classification of 1 (ASA range: 1 to 3), and all Tonnis grades were either 0 or 1. The mean PSQI global score for all patients was 8.46 ± 4.35 (PSQI range: 0 to 21), indicating poor sleep quality. The adaptive LASSO-penalized least squares multiple linear regression revealed that HOOS Pain, SF-12 Role Emotional, and SF-12 Mental Health significantly predicted Sleep Quality (Adjusted R2 = 0.4041). Sleep quality improved as pain, emotional problems, and mental health improved.

**Conclusion:**

Patients with symptomatic FAI syndrome and AD have poor sleep quality. Worsening pain from a patient’s hip pathology is associated with poor sleep, even prior to the onset of osteoarthrosis of the hip. Patients presenting with hip pain from FAI syndrome and AD should be screened for sleep disturbance and may benefit from a multidisciplinary treatment approach.

## Background

Femoroacetabular impingement (FAI) syndrome is characterized by abnormal contact forces of the hip with an incidence of the cam morphology predicted to be approximately 14–68% of the general population based on the inclusion criteria, and there is a predominance of young, athletic patients affected [1–6]. The presence of FAI syndrome has been hypothesized to contribute to hip degeneration and future osteoarthritis [7]. Acetabular dysplasia (AD) is a condition in which the acetabulum is shallow and does not provide sufficient coverage of the femoral head [8]. There is an estimated prevalence of 0.1% in the adult population, and it is a known precursor to osteoarthritis, with evidence of acetabular dysplasia in 20–40% of patients with osteoarthritis of the hip [9–11]. Both hip pathologies are associated with hip pain, decreased quality of life, and morbidity in an often-young active population [12, 13]. Nocturnal pain and poor sleep are often reported, but little is known on the variables that predict sleep disturbance in these patients.

Nocturnal pain has been shown to affect sleep quality, although there is limited evidence of such effects in FAI syndrome and AD. Effects of other musculoskeletal pathologies on sleep quality have been thoroughly studied. A recent study conducted by Khazzam et al. has shown a significant relationship between sleep disturbance and shoulder pain [14]. A study conducted by Tang et al. showed 53% of patients with chronic back pain reported sleep disturbances consistent with clinical insomnia [15]. Decreases in sleep quality have been linked to overall decreases in quality of life, increases in rates of depression and anxiety, and many metabolic disturbances such as diabetes and obesity [16–18]. Long-term impacts of poor sleep quality have shown to have a particularly negative outcome in young, healthy patients [16]. Given the prevalence of these two hip diagnoses in the young adult population, sleep is an especially important factor to be considered because of the significant impact on health and quality of life.

The purpose of this study was to assess how hip pain from FAI syndrome and AD, as well as other patient factors, in a young adult population with symptomatic hip pain affect self-reported sleep quality. We hypothesize that patients with symptomatic FAI syndrome and AD are susceptible to poor sleep quality.

## Methods

### Participants

We prospectively evaluated 115 patients presenting to a comprehensive orthopedic hip clinic in Dallas, USA with a chief complaint of hip pain, and who were diagnosed with either FAI syndrome (*n* = 62) or AD (*n* = 53), at a single academic medical center between November 2016 and May 2017. Diagnoses were made by clinical and radiologic evaluation by the treating orthopaedic surgeon. FAI syndrome was diagnosed by clinical and radiographic evaluation [19]. Diagnosis of FAI syndrome was based on evidence of restricted range of motion and anterior impingement test in patients with radiographic evidence of alpha angle greater than 55 or lateral center edge angle greater than 38 [1, 11, 20–22]. Patients that presented with hip pain and radiographic evidence of femoral head uncovering and a lateral center-edge angle less than 20 degrees were diagnosed with symptomatic AD [23–28]. Patients with concurrent diagnoses, such as hip osteoarthritis or previous hip surgeries, were excluded from the study. A total of 106 patients were excluded on the basis of an osteoarthritis diagnosis. All patients included in the study were pre-treatment and pre-surgical patients treated by the senior author, fellowship trained in hip preservation.

All 115 patients voluntarily completed a comprehensive set of IRB-approved questionnaires that assessed hip function, sleep quality, pain, and overall health. The sample size established was a convenience sample consisting of patients presenting to the clinic with the diagnoses in question. In particular, patients completed questionnaires that included the Hip disability and Osteoarthritis Outcome Score (HOOS), Hip Outcome Score (HOS) scale, Modified Harris Hip Score (mHHS) scale, UCLA Activity scale, International Hip Outcome Tool (iHOT), Visual Analogue Pain Scale (EQ-VAS), Short Form Health Survey (SF-12), and the Pittsburgh Sleep Quality Index (PSQI) [29–36].

### Outcome measure

The primary outcome measure was sleep quality. Sleep quality was measured using the total score on the Pittsburgh Sleep Quality Index (PSQI) Scale [29]. The PSQI is a standardized measure of sleep quality. It consists of 19 self-reported questions regarding various aspects of sleep quality during the previous month from the time of assessment [29]. The final scores of the PSQI consist of seven component scores, with each component scored 0 (no sleep difficulty) to 3 (severe sleep difficulty). The seven PSQI component scores were summed to produce a total, or global, score that ranged from 0 to 21. Higher PSQI Total (global) Scores indicate worse sleep quality [29].

### Potential predictor variables

An initial pool of 39 characteristic variables was selected for analysis as potential predictors of Sleep Quality. The pool of potential predictors, which was selected a priori, included: Diagnosis group (FAI syndrome vs. AD), sex, age (years), BMI (kg/m^2^), ASA physical status classification system (ASA 1 – ASA 4), Tonnis grade (grades 0–3), EQ-VAS (overall health state), patient function level (scored 0–100%, higher score indicated greater normal function level), International Hip Outcome Tool (iHOT) total score (measure of quality of life), Modified Harris Hip score (left and right hip), Hip outcome subscale scores (activity of daily living, sports), UCLA activity score, VAS pain level (averaged score), SF-12 subscale scores (general health, physical functioning, role functioning, bodily pain, vitality, role emotional, mental health, and social functioning; each of the SF12 subscales was scored so that a higher score indicated greater functioning), HOOS subscale scores (pain, symptoms, sports and recreational, quality of life, and activity of daily living). Each HOOS subscale score ranged from 0 to 100 and was scored so that a higher score indicated greater functioning. The following comorbidities were also assessed (yes/no) and included as potential predictors: current smoking status, heart disease, high blood pressure, lung disease, diabetes, ulcer or stomach disease, kidney disease, depression, obstructive sleep apnea, liver disease, and low back pain. Each of the potential predictors was patient self-reported and measured during the patients’ most recent orthopaedic clinic visit.

### Statistical analysis

Demographic and clinical characteristics for the sample of 115 patients were described using the sample mean and standard deviation for continuous variables and the frequency and percentage for categorical variables. To identify differences on these characteristics between those diagnosed with FAI syndrome vs. AD, we implemented the two-independent sample t-test with the Satterthwaite method for unequal variances (for continuous variables) and Fisher’s exact test (for categorical variables).

Next, to utilize the maximum potential of the data, we followed the recommendations of Shomaker et al. and carried out both multiple imputation of missing data and the bootstrap [37]. Starting with the initial pool of 39 variables, a filtering process was used to identify a subset of variables that seemed to contain predictive power. The process was implemented using the adaptive LASSO-penalized variable selection method, with the Shwarz Bayesian information criterion, in the context of a multiple linear regression model for the outcome of sleep quality that was based on 10,000 bootstrap samples [38]. The goal of the adaptive LASSO-penalized linear regression was to select a parsimonious and well-fitting subset of potential predictors of sleep quality by performing simultaneous variable selection and parameter estimation. This is done by optimizing a penalized least squares criterion that expresses a balance between good fit and parsimony. Finally, the one-way ANCOVA was used to compare the two diagnosis groups (FAI syndrome vs. AD) on the measure of sleep quality, while controlling for age, sex, as well as the predictors that emerged from the adaptive LASSO-penalized regression. Least squares means (LSM) was estimated as part of the ANCOVA model. Cohen’s *d* was calculated to estimate the effect size for the between-subjects group effect.

Statistical analyses were carried out using SAS software, version 9.4 (SAS Institute, Inc., Cary, NC, USA). The level of significance was set at α = 0.05 (two-tailed) and we implemented the False Discovery Rate (FDR) procedure, where applicable, to control false positives over the multiple tests.

## Results

### Participant characteristics

Of the total sample of 115 patients, 70.43% were female, the mean age was 34.55 ± 11.66 years (age range = 14 to 58 years), 54% had a diagnosis of FAI syndrome (*n* = 62), and 46% had a diagnosis of AD (*n* = 53). Eighty-nine percent of the patients with a diagnosis of AD and 79% of FAI syndrome reported poor sleep quality (mean PSQI total: 9.00 ± 4.37 and 8.00 ± 4.32). The mean PSQI score of all patients was 8.46 ± 4.35, indicating an average poor sleep quality of the overall patients in the study. Mean scores for HOOS Pain, SF12 Role Emotional, and SF12 Mental Health were 64.02 ± 19.02, 47.37 ± 12.17, and 43.02 ± 11.68, respectively. Demographic and clinical characteristics of the patient cohort are shown in Table 1.

**Table 1:** Demographic and clinical characteristics of the overall sample and by hip diagnosis

Characteristic	Overall Sample (*N* = 115)	Femoroacetabular Impingement (*n* = 62)	Acetabular Dysplasia (*n* = 53)	*p*-value (FDR)
Patient Demographics				
Age, years, M (SD)	34.55 (11.66)	37.50 (11.70)	31.11 (10.71)	0.003 (0.04)
Female Gender, % (n)	70.43% (81)	59.68% (37)	83.02% (44)	0.007 (0.04)
Patient Factors				
BMI, kg/m^2^, M (SD)	26.40 (5.23)	26.43 (5.66)	26.37 (4.74)	0.95 (0.99)
Tonnis Grade 0, % (n)	53.04% (61)	53.23% (33)	52.83% (28)	0.99 (0.99)
Tonnis Grade 1, % (n)	46.96% (54)	46.77% (29)	47.17% (25)	0.99 (0.99)
ASA Classification 1, % (n)	76.52% (88)	72.58% (45)	81.13% (43)	0.60 (0.87)
Current Smoker, % (n)	5.22% (06)	9.68% (06)	0.00% (53)	0.03 (0.10)
iHOT (Quality of Life), M (SD)	46.48 (22.72)	51.14 (24.11)	41.03 (19.84)	0.01 (0.04)
UCLA Activity Score, M (SD)	6.50 (2.55)	7.13 (2.74)	5.77 (2.11)	0.003 (0.04)
SF-12 General Health, M (SD)	48.62 (10.21)	49.15 (9.76)	47.99 (10.77)	0.54 (0.82)
SF-12 Role Emotional, M (SD)	47.37 (12.17)	47.69 (12.13)	47.01 (12.32)	0.76 (0.96)
SF-12 Mental Health, M (SD)	43.02 (11.68)	44.77 (11.97)	40.96 (11.08)	0.08 (0.19)
Modified Harris Hip Score, M (SD)	74.38 (19.68)	77.27 (18.53)	71.01 (20.42)	0.09 (0.20)
Patient Function Level, M (SD)	56.47 (27.71)	57.98 (27.30)	54.71 (28.34)	0.53 (0.82)
EQ-VAS, M (SD)	72.34 (17.87)	72.75 (17.67)	71.86 (18.26)	0.79 (0.96)
HOOS ADL, M (SD)	74.56 (18.57)	77.70 (18.58)	70.89 (18.05)	0.05 (0.14)
HOOS QOL, M (SD)	39.24 (21.61)	44.15 (21.43)	33.49 (20.55)	0.007 (0.04)
HOOS Pain, M (SD)	64.02 (19.02)	67.90 (18.60)	59.48 (18.65)	0.01 (0.04)
HOS ADL, M (SD)	70.66 (19.06)	73.64 (19.52)	67.16 (18.06)	0.07 (0.18)
HOS Sports, M (SD)	56.52 (22.93)	60.32 (24.85)	52.06 (19.77)	0.05 (0.14)
PSQI Total Score, M (SD)	8.46 (4.35)	8.00 (4.32)	9.00 (4.37)	0.22 (0.42)
Patient Comorbidities, % (n)				
Low Back Pain	49.57% (57)	59.68% (37)	37.74% (20)	0.02 (0.08)
Depression	16.52% (19)	17.74% (11)	15.09% (08)	0.80 (0.96)
High Blood Pressure	8.70% (10)	11.29% (07)	5.66% (03)	0.33 (0.56)
Lung Disease	7.83% (09)	11.29% (07)	3.77% (02)	0.17 (0.35)
Ulcer/Stomach Disease	5.22% (06)	6.45% (04)	3.77% (02)	0.68 (0.94)
Obstructive Sleep Apnea	4.35% (05)	4.84% (03)	3.77% (02)	0.99 (0.99)
Diabetes	2.61% (03)	4.84% (03)	0.00% (00)	0.24 (0.43)
Heart Disease	1.74% (02)	1.61% (01)	1.89% (01)	0.99 (0.99)

Note. *M* Sample Mean, *SD* Standard Deviation. *P*-value (2-tailed) associated with the test of group differences (femoroacetabular impingement vs. acetabular dysplasia) on each characteristic. *FDR* False Discovery Rate. All characteristics were self-reported by the patient

### Orthopedic diagnosis and sleep quality

The one-way ANCOVA revealed no significant diagnosis group (FAI syndrome vs. AD) main effect on sleep quality (F = 0.01; df = 1108; *p* = 0.9315), while controlling for age, sex, HOOS Pain, SF12 role emotional, and SF12 mental health. Although the pattern of the adjusted least squares means (LSM) revealed that PSQI Total (global) scores were not significantly different between the FAI and AD diagnosis groups (FAI LSM = 8.4891, SE = 0.4562 vs. AD LSM = 8.4278, SE = 0.4978; Cohen’s *d* = 0.0162), both diagnosis groups had global LSM PSQI scores above 5, indicating poor sleep quality.

#### Predictors of Sleep Quality

**Fig. 1:**
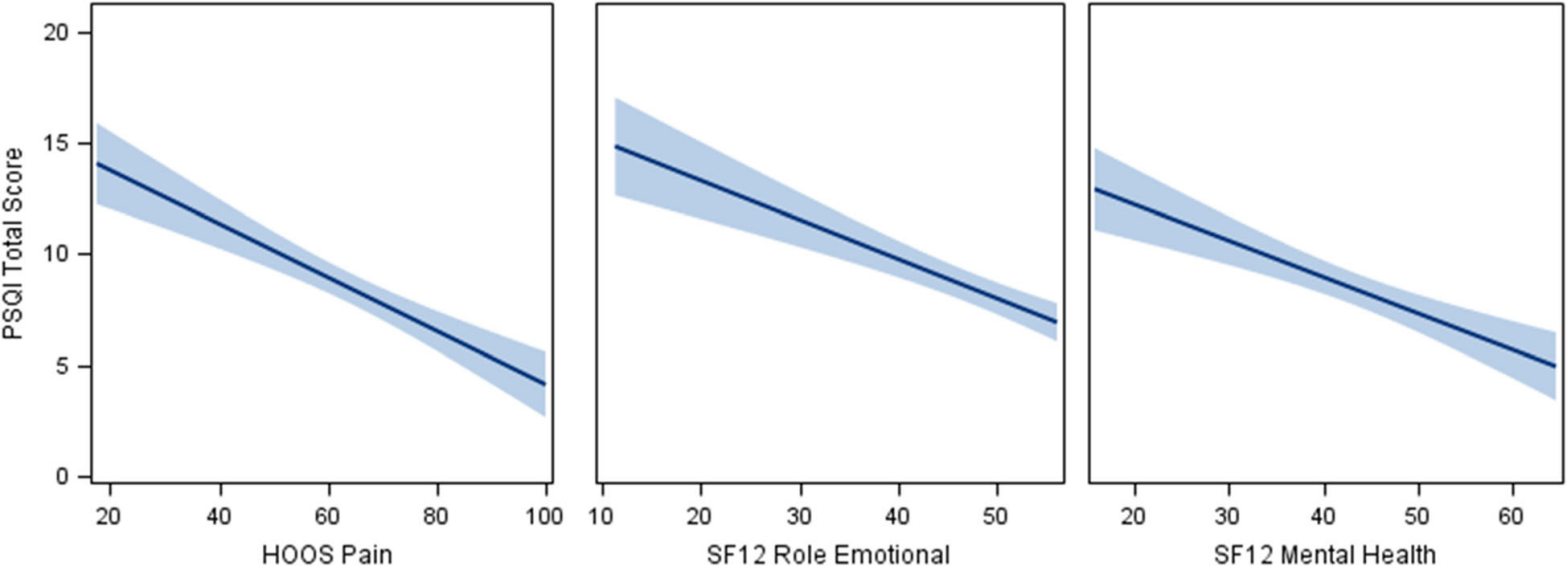
Plot of PSQI Total Score against the selected predictor variables, with a fitted linear regression line and 95% confidence limits. Observed sample, *N* = 115

The subset of predictor variables that were selected from the multiple linear regression, with adaptive LASSO variable selection, for the outcome of sleep quality is reported in Table 2 and Fig. 1.

**Table 2:** Multiple Linear Regression Model for Predictors of Sleep Quality using an Adaptive LASSO-penalized variable selection method

Bootstrapped Adaptive LASSO Parameter Estimates						
Model Outcome and Predictor Variables^a^	Mean Estimate	SD	95% CI	Standardized Estimate	Adjusted R^2^	VIF
PSQI Total					0.4041	
Intercept	20.2082	1.6311	17.0903 to 23.4622	0		0
HOOS Pain	−0.0794	0.0194	−0.1150 to −0.0430	−0.3609		1.1553
SF12 Role Emotional	−0.0817	0.0292	− 0.1405 to − 0.0320	−0.2635		1.3337
SF12 Mental Health	−0.0653	0.0293	−0.1263 to − 0.0199	−0.1678		1.3234

Note. The adaptive LASSO estimates were based on 10,000 bootstrap samples of the model; Mean Estimate = bootstrap parameter estimate (regression coefficient); *SD* Standard deviation of the mean parameter estimate; 95% CI for the mean parameter estimate; For the 95% CI that does not contain zero (0), the respective mean parameter estimate is statistically significant at alpha = 0.05 (two-tailed); Standardized Estimate = bootstrap standardized regression coefficient; Adjusted R-squared is the model R-squared based on the adaptive LASSO-penalized variable selection; *VIF* Variance Inflation Factor. Observed sample, *N* = 115. ^a^Predictor variables were selected from a pool of 39 potential predictor variables via the adaptive LASSO-penalized variable selection method (which performs simultaneous variable selection and parameter estimation) in the context of a linear regression model that was based on 10,000 bootstrap samples. *PSQI Total* Pittsburgh Sleep Quality Index Total Score, *HOOS Pain* Hip disability and Osteoarthritis Outcome Score (Pain subscale), *SF12* Short Form Health Survey (subscales for Role Emotional and Mental Health)

We reported the averaged LASSO-penalized parameter estimates and standard deviation that were based on 10,000 bootstrap samples of the multiple linear regression models along with the 95% bootstrap confidence interval. For the 95% CI that did not contain zero, the respective mean parameter estimate was statistically significant at alpha = 0.05 (two-tailed). As shown in Table 2, the adaptive LASSO-penalized least squares multiple linear regression revealed that HOOS Pain, SF12 Role Emotional, and SF12 Mental Health significantly predicted Sleep Quality (Adjusted R^2^ = 0.4041). Sleep quality improved as pain, role emotional, and mental health improved. Of the selected predictor variables from the adaptive LASSO-penalized multiple linear regression model (Table 2), the standardized parameter estimates revealed that HOOS Pain can be interpreted as having a greater magnitude of relative importance in the expected relationship with Sleep Quality (standardized beta = − 0.3609).

## Discussion

The findings of this study showed 83% of patients presenting with FAI syndrome and AD reported poor sleep quality. Significant predictors of sleep quality in our patient cohort included the HOOS pain, SF-12 survey role limitations due to emotional problems, and SF-12 survey mental health problems. Indeed, we found that sleep quality was better with less pain, role emotional, and mental health scores. The HOOS Pain had the greatest magnitude of importance when predicting sleep quality. Given that a PSQI score of 5 or greater indicates poor sleep quality, the average score of 8.46 in our cohort shows a marked decline in sleep quality compared to the general population. Although we found no significant difference in sleep quality when comparing FAI syndrome to AD, both diagnosis groups had global PSQI scores above 5, indicating poor overall sleep quality. We investigated several potential confounding variables to isolate the patient’s hip pain as the true reason for sleep disturbance, and the variables remained independent predictors of sleep. This supports symptomatic FAI syndrome and AD as major factors in determining sleep quality.

The effects of other musculoskeletal pathologies on sleep quality have been thoroughly studied. Previous research has shown that patients with disorders such as osteoarthritis and chronic low back pain suffer from considerably reduced sleep quality [39, 40]. Rhon et al. described in a 2018 study that the incidence of sleep disorders rose significantly following elective hip arthroscopy when compared to preoperative sleep status [41]. This comorbidity is often overlooked as a risk of hip intervention. However, a study by Gong et al. showed that clinical interventions targeting sleep have shown to improve quality of life and patient satisfaction following total knee arthroplasty [42]. We have now shown that hip pain secondary to FAI syndrome and AD also causes sleep disturbance. Sleep quality is a potential area of improvement in patient outcomes in FAI syndrome and AD.

Few studies have looked at the association of pain caused by FAI syndrome and AD with that of poor sleep [43, 44]. A 2017 study by Prather et al. showed significant disorders of sleep in young and middle-aged patients with hip pain [44]. While their findings support our work, Prather et al. did not employ validated hip outcome scales to quantify the effect of patient-reported symptoms due to hip pathology. To our knowledge, the current study is the first to utilize validated patient questionnaires to determine predictors of sleep quality in patients with FAI syndrome and AD. In our patient population with FAI syndrome and AD, even though young with minimal comorbidities, sleep disturbance is found to a great degree.

The validated patient reported outcome measures used in the current study to measure hip function, sleep quality, pain, and overall health are easy to administer in an outpatient setting and should be utilized to screen for sleep quality. They can be used to routinely to evaluate change in sleep quality in order to assess deficits before possible detrimental effects. This has significant implications for a patient’s mental and emotional health, quality of life, and overall wellness, and is particularly important in our patient cohort given it consists mainly of young, healthy patients. Young patients are at risk for long-term morbidity as a consequence of decreased sleep quality. Addressing the problem of nocturnal pain causing poor sleep may reduce the risk of impairment. Orthopaedic physicians should spend time assessing patients’ sleep quality as well as counseling them on possible adverse effects of hip pain on sleep [45]. A study conducted by Kunze et al. in 2019 showed an improvement in sleep quality following hip arthroscopy for patients with FAI syndrome [43]. In patients who are not ideal candidates for this treatment, other treatments specifically for sleep disturbance to be considered include education on sleep hygiene, ideal positioning, and sleep medicinal aids.

Limitations in this study include the subjective nature of self-reported patient questionnaires. This could have led to inconsistencies in reporting among individual patients. In addition, the lack of standardized variables within the study such as polysomnography and other sleep studies furthers the subjective nature of the data presented here. However, given the subjective nature of pain and sleep quality, it is arguably more clinically significant to obtain self-reported measures in regard to overall wellness. Another limitation in this study is the lack of data reporting the patient cohort’s sleep quality prior to the diagnosis of FAI syndrome or DDH. Additionally, these patients were not compared with age-matched controls, although historically this cohort, consisting of young patients with minimal comorbidities, is found to have less pathologic sleep [46]. All patients in the study came from one orthopedic specialty clinic, so results may not generalize to the all orthopedic patients with varying diagnoses of pain. Further important areas of study would be to follow patients with sleep disturbance throughout their treatment and assess sleep quality changes over time.

## Conclusion

In conclusion, we found that patients with hip pain from that of FAI syndrome and AD are susceptible to poor sleep quality. We also found that sleep quality was better with higher scores indicating lower pain, fewer role limitations due to emotional problems, and improved mental health. Patients presenting with hip pain from FAI syndrome and AD should be screened for sleep disturbance and may benefit from a multidisciplinary treatment approach. Future research should look into treatment of these specific hip pathologies and its effect on sleep quality. It is important to note whether treatment of FAI syndrome and AD has an effect on self-reported sleep quality using the same measurement tool as this study. Different modes of management for each condition can be compared to assess which option is better suited to improve overall sleep quality.

## Data Availability

The data generated for this study is not publicly available in order to protect patient privacy. The corresponding author may provide this information on reasonable request.

## Abbreviations

AD: Acetabular dysplasia
EQ-VAS: Visual Analogue Pain Scale
FAI: Femoroacetabular impingement
HOOS: Hip disability and Osteoarthritis Outcome Scor
HOS: Hip Outcome Score
iHOT: International Hip Outcome Tool
mHHS: Modified Harris Hip Score
PSQI: Pittsburgh Sleep Quality Index
SF-12: Short Form Health Survey
